# Antibiotic resistance and factors associated with colonization dynamics of *Staphylococcus aureus* and *Streptococcus pneumoniae* in healthy children in Lima, Peru

**DOI:** 10.1017/S0950268825100277

**Published:** 2025-09-04

**Authors:** Valeria Mariana Li Valverde, Paulo Cesar Aguirre Castañeda, Brayan E. Gonzales, Franco Castillo-Tokumori, Jorge E. Vidal, Theresa J. Ochoa

**Affiliations:** 1 Facultad de Medicina, Universidad Peruana Cayetano Heredia, Lima, Peru; 2 Instituto de Medicina Tropical Alexander von Humboldt, Universidad Peruana Cayetano Heredia, Lima, Peru; 3 Grupo Peruano de Investigación en Neumococo (GPIN), Lima, Peru; 4 Department of Cell and Molecular Biology, Center for Immunology and Microbial Research, University of Mississippi Medical Center, Jackson, MS, US

**Keywords:** co-colonization, pneumococcal conjugate vaccines, *Streptococcus pneumoniae*, *Staphylococcus aureus*

## Abstract

Pneumococcal conjugate vaccines (PCVs) have influenced population dynamics of *Streptococcus pneumoniae* in the nasopharynx and may have contributed to increased *Staphylococcus aureus* colonization. This study assessed the prevalence of colonization, antibiotic resistance patterns, and associated risk factors for colonization and co-colonization of *S. aureus* and *S. pneumoniae* in healthy Peruvian children post-PCV introduction. Nasopharyngeal swabs from children <24 months were collected in five hospitals in Lima (2018–2019). Microbiological identification and antibiotic susceptibility tests were performed, and multinomial regression evaluated factors influencing colonization. Among 894 children, 19.7% were colonized with *S. aureus*, 20.3% with *S. pneumoniae*, and 2.9% co-colonized. Of the 176 *S. aureus* strains isolated, 1.7% were methicillin resistant and 20.5% were clindamycin resistant; no resistance to trimethoprim-sulfamethoxazole (SXT) was found. Among 182 *S. pneumoniae* strains isolated, 48.9% were resistant to macrolides, 74.7% to SXT; no resistance to penicillin was found. Breastfeeding and vaccination with PCV13 were associated with a reduced prevalence of *S. aureus* colonization, while vaccination with PCV13 increased the prevalence of *S. pneumoniae* colonization, mainly by non-vaccine serotypes. This study highlights the need to continue monitoring the changes in colonization dynamics and antimicrobial resistance patterns after vaccine introduction, to guide empirical therapy and future vaccine strategies.

## Introduction

Pneumococcal conjugated vaccines (PCV) represent a global strategy to reduce the burden of diseases caused by *S. pneumoniae* (pneumococcus). However, with their widespread implementation, concerns have arisen about the potential increase in *S. aureus* infections [1]. *S. aureus* is an opportunistic organism that colonizes mucous membranes and the skin of 20%–80% of the human population without symptoms [2]. It is a common cause of bacterial infections both in the community and hospital settings. In 2024, the World Health Organization (WHO) updated the list of the main drug-resistant pathogens, including *S. aureus* methicillin-resistant and *S. pneumoniae* macrolide-resistant, as bacteria with high and medium priority, respectively [3].

Following the introduction of PCVs, there has been a replacement of pneumococcal serotypes with an increase in the prevalence of non-vaccine serotypes (not included in the PCVs) that are clinically relevant, such as serotype 19A. The first clinical trial evaluating PCV7 and its impact on *S. aureus* colonization reported an increase in the frequency of colonization after receiving two doses [4]. Furthermore, an inverse association between *S. pneumoniae* vaccine serotypes (serotypes included in the PCVs, PCV-serotypes) and *S. aureus* is also reported [4–6], suggesting that colonization dynamics involve a complex interplay of bacterial and host factors.

Describing the patterns of antibiotic resistance in community-acquired *S. aureus* is important because antibiotic treatment with a drug to which *S. aureus* is resistant promotes colonization and, consequently, the development of potentially serious infections [7]. Additionally, there is no information on the rate of methicillin-resistant *S. aureus* (MRSA) colonizing healthy Peruvian children. The study aimed to assess the antibiotic resistance of *S. aureus* in nasopharyngeal carriers under 24 months of age between 2018 and 2019 in Lima, Peru, determine the prevalence of carriage and co-colonization with *S. pneumoniae*, and identify potential factors associated with colonization status.

## Methods

### Study design and population

We used samples from a cross-sectional multicentre study conducted to evaluate changes in pneumococcal serotypes after PCV13 introduction [8]. The previous study enrolled 1,000 healthy children between 2 and 24 months of age who attended the well-child outpatient or the vaccination services of five hospitals in Lima, Peru. Only 894 children were enrolled from the previous study since their parents or caregivers provided consent to use their data and samples for future studies.

The main study defined a ‘healthy child’ as any child who attended the growth, development, and/or immunization outpatient clinic at Cayetano Heredia Hospital, Instituto de Salud del Niño Sede Breña, Edgardo Rebagliati Martins Hospital, Hospital Docente Madre-Niño San Bartolome, or Daniel Alcides Carrion Hospital, and whose parent or caregiver reported that, at the time of enrolment, the child did not suffer from any major illness such as pneumonia, sepsis, bacteraemia, or meningitis/encephalitis. Children might have had mild respiratory infections such as rhinorrhea, mild cough, sneezing, or a temperature of 38.0 to 38.5 °C at enrolment [8].

### Sample collection

Nasopharyngeal swab samples were taken and placed in STGG transport medium (skim milk, tryptic soy broth, glucose, glycerol) and transported in coolers within 8 h to the Paediatric Infectious Diseases Laboratory (LIP) at Cayetano Heredia University (the central laboratory of the main study), for subsequent microbiological analysis.

### Laboratory studies

As part of the main study, the swabs were placed in 3 ml of THY medium (Todd-Hewitt Broth with 0.5% yeast extract and 5% rabbit serum) and incubated for 4 to 6 h at 37 °C with 5.0% CO_2_. The bacterial growth was cultured on blood agar (Trypto-Casein Soy Agar enriched with 5% sheep blood) under the same previously described conditions for 24 h. *S. pneumoniae* isolates were identified through colony morphology, the presence of alpha haemolysis, Gram staining, bile solubility, and optochin sensitivity [9]. *S. aureus* isolates were identified through the bacterial growth obtained in THY medium, plated on Mannitol Salt Agar, and with the Gram staining, catalase, and coagulase tests [10]. The ATCC 25923 and ATCC 43300 strains were used as controls for *S. aureus* and D39/NCTC 7466 for *S. pneumoniae.* Pneumococcal serotype was determined by whole genome sequencing (WGS).

### Susceptibility testing

Antibiotic susceptibility of *S. aureus* to erythromycin, clindamycin, cefoxitin (to determine methicillin resistance) [11], penicillin, and trimethoprim-sulfamethoxazole (SXT) were determined by Kirby Bauer method using antibiotic disks (Oxoid Ltd., Basingstoke, Hans, UK), as well as *D*-Test to identify clindamycin resistance induced by erythromycin on Mueller Hinton agar and incubated for 24 h at 37 °C [11]. Antibiotic susceptibility of *S. pneumoniae* to azithromycin, clindamycin, ceftriaxone, penicillin, and SXT was determined by MIC using E-test® (AB Biodisk, Solna, Sweden) on Mueller Hinton agar with 5% sheep blood and incubated for 24 h at 37 °C in an atmosphere of 5% CO_2_. Interpretation was carried out according to the Clinical and Laboratory Standards Institute (CLSI) guidelines [11].

### Statistical analysis

Bivariate analyses were performed to determine the associations between the dependent variable (colonization status), the independent variable, and covariates of interest using parametric and non-parametric tests such as χ^2^ and Fisher’s exact test (variables that meet or do not meet Cochran’s rules, respectively), ANOVA (variables with normality distribution), and Kruskal–Wallis (variables without normality distribution). Multinomial regressions were conducted to determine associations with the colonization status of *S. pneumoniae*, *S. aureus*, and co-colonized subjects. Additionally, Poisson regression with robust variances was performed to explore the association between children colonized by *S. pneumoniae* and *S. aureus* (this model only considered these two variables independently and not as a polytomous variable as used in the multinomial regression model). In the multivariate analysis, covariates were included as identified in the literature and by the clinical experience of previous studies that resulted in statistical significance in our bivariate analyses, as identified in the Directed Acyclic Graph (DAG) (Supplementary Figure S1). The category ‘not colonized’ was considered the comparator group. All measures of association were the prevalence ratio (PR) (this was approximated using risk ratios (RR) and adjusted using the estimation option ‘rrr’ (multinomial model) and robust standard errors (Poisson model)) due to the study design. The analyses were performed in Stata/SE version 18.0 (StataCorp LP, College Station, TX), considering a 95% confidence interval, and the statistical significance level was set a *p* < 0.05.

### Ethical aspects

This study was approved by the Institutional Review Board of Cayetano Heredia University (Lima, Peru)

## Results

A total of 894 samples from healthy children enrolled between 2018 and 2019 were analyzed; 19.7% were positive only for *S. aureus*, 20.3% were positive only for *S. pneumoniae*, and 2.9% had co-colonization with both bacteria (Table 1). The mean age of the children was 11.1 months; 20.9% were preterm, and 37.2% completed the recommended 6 months of exclusive breastfeeding. Mild respiratory illness, characterized by mild cough, rhinorrhea, sneezing, or a temperature between 38°C and 38.5°C, was presented in 36.3%. PCV vaccination coverage, with at least one dose, was 89.4% (Table 1).

**Table 1.** Characteristics of children included in the study (*N* = 894)^a^

Characteristics	*N* (%)	Colonization status				*p*
		Non-colonized	Only *S. aureus*	Only *Streptococcus pneumoniae*	Co-colonized	
		*N* = 562	*N* = 150	*N* = 156	*N* = 26	
		*n* (%)	*n* (%)	*n* (%)	*n* (%)	
Sex (male)	435 (48.7)	266 (47.4)	77 (51.3)	74 (47.4)	18 (69.2)	0.153^f^
Age (months)^b^	11.1 ± 6.4	11.3 ± 6.3	9.9 ± 7.0	11.6 ± 6.0	11 ± 6.7	0.089^g^
Preterm birth	182 (20.9)	120 (22.0)	34 (23.0)	26 (17.2)	2 (7.7)	0.189^f^
Breastfeeding duration (months)^b^	8.0 (4–14)	8.0 (5–14)	6.0 (3–13)	9.5 (5–14)	7.5 (4–15)	0.059^h^
Exclusive breastfeeding (6 months)	329 (37.2)	216 (39.0)	35 (23.5)	68 (43.9)	10 (38.5)	0.001^f^
Day-care centre attendance	53 (6.0)	34 (6.1)	9 (6.0)	9 (5.8)	1 (4.0)	0.977^f^
Living with children <6 years old	348 (39.6)	195 (35.2)	63 (42.6)	78 (51.7)	12 (46.2)	0.002^f^
Mild respiratory illness^c^	322 (36.3)	189 (33.9)	45 (30.0)	78 (50.7)	10 (38.5)	<0.001^f^
Prior hospitalization^d^	57 (6.4)	41 (7.3)	11 (7.3)	4 (2.6)	1 (3.9)	0.162^f^
Antibiotics use^d^	247 (27.8)	167 (29.9)	36 (24.0)	40 (25.8)	4 (15.4)	0.205^f^
Seasonal distribution^e^						0.773^f^
Summer	342 (38.2)	209 (37.2)	58 (38.7)	64 (41.0)	11 (42.3)	
Fall	244 (27.3)	151 (26.9)	39 (26.0)	46 (29.5)	8 (30.8)	
Winter	167 (18.7)	107 (19.0)	26 (17.3)	29 (18.6)	5 (19.2)	
Spring	141 (15.8)	95 (16.9)	27 (18.0)	17 (10.9)	2 (7.7)	
Immunized with at least one PCV13 dose	750 (89.4)	479 (90.6)	110 (78.0)	139 (95.9)	22 (91.7)	<0.001^f^
Number of PCV doses						<0.001^f^
Not immunized	89 (10.7)	50 (9.5)	31 (22.6)	6 (4.1)	2 (8.3)	
One dose	122 (14.7)	71 (13.5)	25 (18.3)	21 (14.5)	5 (20.8)	
Two doses	321 (38.6)	205 (39.0)	38 (27.7)	71 (49.0)	7 (29.2)	
Three doses	300 (36.0)	200 (38.0)	43 (31.4)	47 (32.4)	10 (41.7)	
PCV13 serotype	36 (19.8)	–	–	29 (18.8)	5 (19.2)	1.000^i^

a Some variables may add <894 due to missing data.

b Median (IQR).

c Rhinorrhea, mild cough, sneezing, temperature ≤ 38.5 °C.

d Within 3 months preceding sampling.

e Season of swab collection.

f χ^2^ test.

g ANOVA.

h Kruskal–Wallis test.

i Fisher’s exact test.

### Characteristics and factors associated with colonization

According to the multivariate analysis, the frequency of colonization by *S. aureus* alone was 0.59 times lower (95% CI: 0.37–0.94; *p* = 0.028) among those who completed exclusive breastfeeding and 0.48 times lower (95% CI: 0.26–0.87; *p* = 0.017) among those who received at least one dose of PCV13 (Table 2). The frequency of colonization by *S. pneumoniae* alone was 3.00 times higher (95% CI: 1.17–7.69; *p* = 0.022) among those who received at least one dose of PCV13, 1.95 times higher (CI: 1.32–2.90; *p* = 0.001) among those with mild respiratory illness, and 1.99 times higher (95% CI: 1.35–2.90; p = 0.001) among children living with other children. In contrast, the prevalence was 0.29 times lower (95% CI: 0.09–0.99; *p* = 0.048) among those who had been previously hospitalized. No significant association was found between co-colonization (*S. pneumoniae* + *S. aureus*) and PCV13 (*p* = 0.521), except for sex, where the prevalence of co-colonization prevalence was 2.50 times higher in males than in females (95% CI: 1.00–6.21; *p* = 0.049, (Table 2)).

**Table 2.** Factors associated with nasopharyngeal colonization in Peruvian children (multivariate analysis)

Characteristics	Colonization status								
	Only *S. aureus*			Only *Streptococcus pneumoniae*			Co-colonized		
	aPR	IC 95%	*p*	aPR	IC 95%	*p*	aPR	IC 95%	*p*
Sex									
Female	Ref.			Ref.			Ref.		
Male	1.08	0.73–1.58	0.705	1.05	0.71–1.55	0.802	2.50	1.00–6.21	0.049
Age (months)	1.00	0.97–1.04	0.789	0.98	0.95–1.02	0.275	0.97	0.90–1.05	0.523
Exclusive breastfeeding (6 months)									
No	Ref.			Ref.			Ref.		
Yes	0.59	0.37–0.94	0.028	1.22	0.80–1.87	0.354	0.98	0.38–2.56	0.969
Mild respiratory									
No	Ref.			Ref.			Ref.		
Yes	0.83	0.55–1.26	0.38	1.95	1.32–2.90	0.001	1.42	0.59–3.40	0.428
Day-care centre attendance									
No	Ref.			Ref.			Ref.		
Yes	0.98	0.41–2.37	0.966	0.53	0.20–1.45	0.216	0.72	0.09–5.84	0.755
Living with children <6 years old									
No	Ref.			Ref.			Ref.		
Yes	1.35	0.91–1.99	0.134	1.99	1.35–2.94	0.001	1.46	0.62–3.43	0.385
Antibiotics use									
No	Ref.			Ref.			Ref.		
Yes	0.83	0.53–1.32	0.437	0.63	0.40–1.01	0.053	0.41	0.12–1.42	0.158
Prior hospitalization									
No	Ref.			Ref.			-		
Yes	0.76	0.34–1.70	0.507	0.29	0.09–0.99	0.048	-	-	-
Immunized with at least one PCV13 dose									
Non-vaccinated	Ref.			Ref.			Ref.		
Vaccinated	0.48	0.26–0.87	0.017	3.00	1.17–7.69	0.022	1.71	0.33–8.81	0.521

*Note:* Multivariate analysis using robust (modified) multinomial logistic regression. Non-colonized was the comparator group.

aPR: Prevalence ratio. 95% CI: 95% confidence interval. Ref: Reference category. -: Without observations.

Adjusting for sex, age (months), exclusive breastfeeding (6 months), day-care centre attendance, living with children <6 years old, mild respiratory illness, prior hospitalization, antibiotics use, and immunized with at least one PCV13 dose.

Additionally, children colonized by *S. pneumoniae* exhibited a reduced prevalence ratio (RP: 0.68, 95% CI: 0.46–0.99; *p* = 0.047) for colonization by *S. aureus*, suggesting an inverse association between the two potentially pathogenic species. A similar trend was observed with non-vaccine serotypes (RP: 0.69; 95% CI: 0.44–1.06; *p* = 0.087), although this finding did not reach statistical significance. In contrast, no association was identified with vaccine serotypes (RP: 0.70; 95% CI: 0.28–1.79; *p* = 0.461) (Supplementary Table S1). Information about pneumococcus serotypes of this series is described in a previous publication of our group [8].

### Antibiotic susceptibility

We isolated *S. aureus* strains from the nasopharynx of 176 children (Table 1), with only three strains (1.7%) classified as MRSA based on the CLSI-recommended test of resistance to cefoxitin. In contrast, the majority of these strains (89.2%) were resistant to penicillin. Regarding macrolide resistance, 59 (33.5%) *S. aureus* strains were non-susceptible to erythromycin, while 36 strains (20.5%) were non-susceptible to clindamycin. The resistance to clindamycin was also evaluated through the *D*-test, which was positive in 34/59 erythromycin-resistant isolates (57.6%). Regarding this specific antibiotic, out of 36 strains non-susceptible to clindamycin, 34 showed inducible resistance, with 33 identified as MSSA (methicillin-sensitive *S. aureus*). Among 182 pneumococcal isolates evaluated, all were susceptible to penicillin using the non-meningeal breakpoint. Additionally, 48.9% of the isolates were resistant to the macrolide azithromycin (Table 3).

**Table 3.** Antibiotic resistance of *S. aureus* and *Streptococcus pneumoniae* strains

Antibiotic	*S. aureus* susceptibility (*N* = 176)^a^			*S. pneumoniae* susceptibility (*N* = 182)^b^		
	Resistant	Intermediate	Susceptible	Resistant	Intermediate	Susceptible
	*n* (%)	*n* (%)	*n* (%)	*n* (%)	*n* (%)	*n* (%)
Penicillin^c^	157 (89.2)	2 (1.1)	17 (9.7)	0	13 (7.1)	169 (92.9)
Macrolides^d^	59 (33.5)	2 (1.1)	115 (65.3)	89 (48.9)	13 (7.1)	80 (44.0)
Clindamycin	36^e^ (20.5)	1 (0.6)	139 (78.9)	52 (28.6)	2 (1.1)	128 (70.3)
Cefoxitin	3 (1.7)	0	173 (98.3)	–	–	–
Ceftriaxone^c^	–	–	–	0	5 (2.8)	177 (97.3)
SXT^f^	0	0	176 (100.0)	136 (74.7)	8 (4.4)	38 (20.9)

a *S. aureus* strains isolated from children colonized by only *S. aures* and co-colonized.

b *S. pneumoniae* strains isolated from children colonized by only *S. pneumoniae* and co-colonized.

c Non-meningitis breakpoint for *S. pneumoniae* strains.

d Erythromycin tested by *S. aureus* and azithromycin by *S. pneumoniae.*

e 34 isolates exhibited inducible resistance to clindamycin (D-test positive).

f SXT: trimethoprim-sulfamethoxazole.

Regarding the resistance pattern among *S. aureus* isolates, penicillin resistance was the most frequent (56.3%), while 14 strains (7.9%) were classified as pan-susceptibility. In *S. pneumoniae*, the most common resistance pattern involved azithromycin, clindamycin, and SXT (23.1%), followed by resistance to azithromycin and SXT (16.5%). Notably, 12.1% of pneumococcal isolates demonstrated pan-susceptibility to the five antibiotics evaluated (Table 4).

**Table 4.** Antibiotic resistance patterns *S. aureus* and *Streptococcus pneumoniae* strains isolated from healthy children

Antibiotic resistance patterns	*S. aureus*	*S. pneumoniae*
	*N* = 176	*N* = 182^a^
	*n* (%)	*n* (%)
FOX + ERI + PEN + CLD	2 (1.1)	–
AZM + CLD + SXT	–	42 (23.1)
ERI + PEN + CLD	33 (18.8)	0
AZM + SXT	–	30 (16.5)
ERI + PEN	24 (13.6)	–
ERI/AZM + CLD	1 (0.6)	10 (5.5)
FOX + PEN	1 (0.6)	–
PEN	99 (56.3)	–
SXT	–	64 (35.2)
ERI/AZM	1 (0.6)	7 (3.8)
CLD	1 (0.6)	–
Pansensitive^b^	14 (8.0)	22 (12.1)

Abbreviations: FOX = cefoxitin, ERI/AZM = erythromycin tested by *S. aureus* or azithromycin by *S. pneumoniae*, PEN = penicillin, CLD = clindamycin, SXT = trimethoprim-sulfamethoxazole.

a 7 pneumococcal isolates were intermediate to azithromycin and/or trimethoprim-sulfamethoxazole.

b Pansensitive: Susceptible to cefoxitin, erythromycin, trimethoprim-sulfamethoxazole, penicillin and clindamycin.

## Discussion

This study in Peruvian children observed nasopharyngeal colonization rates of 19.7% for *S. aureus*, 20.3% for *S. pneumoniae* of and 2.9% for co-colonization with both species. Importantly, despite concerns about antibiotic resistance, carriage strains of *S. aureus* and *S. pneumoniae* exhibited high susceptibility to methicillin and penicillin, respectively, in this population. While exclusive breastfeeding and PCV13 vaccination were associated with lower *S. aureus* colonization, PCV13 vaccination was associated with increased *S. pneumoniae* colonization, and an increased risk of colonization was observed in children experiencing mild respiratory illness and living with other children.

The observed *S. aureus* colonization rate of our study is lower than reported in other studies. This discrepancy may be attributed to the age range of our population, as *S. aureus* colonization rates typically exhibit age-related variations. Colonization rates are higher in infants (<6 months), decrease during early childhood, increase again between 6 and 12 years of age, and subsequently decline in adulthood [12, 13]. Previous studies have reported varying *S. aureus* colonization rates in children. For instance, Paraguay reported a higher colonization frequency (30.8%) in 2016 [14], with similar rates in Brazil (31.1%), Colombia (33%), and Japan (28.2%). In contrast, Mexico (59.8%) and Venezuela (56%) showed higher rates [6, 15–18]. A study in children from Cajamarca, Peru, in 2009, reported a lower colonization rate of 12.7% using microbiological methods [10]. These variations in colonization rates across different regions can be attributed to a multitude of factors, including environmental and sociodemographic factors specific to each population [14].

Exclusive breastfeeding and PCV13 vaccination were associated with decreased *S. aureus* colonization, aligning with the known benefits of breastfeeding on gut microbiota development [19–21]. While some studies have suggested an association between PCV13 vaccination and decreased *S. aureus* colonization, this relationship is often attributed to the impact of the vaccine on specific pneumococcal serotypes [4]. However, this mechanism was not observed in our study.

It is well-established that individuals colonized with MRSA have a higher risk of developing MRSA-related infections compared to those colonized with MSSA [22]. Risk factors for MRSA colonization include recent hospitalization, recent antibiotic treatment, and the use of beta-lactam antibiotics within the past 3 months [18]. In our study, the prevalence of MRSA among *S. aureus* carriers was low (1.7%), consistent with the range reported in Latin America and globally (0.2–10%) [23, 24]. In Peru, the reported MRSA prevalence was 0.9% in the general population and 0.3% in the military [23, 25]. In Brazil, MRSA prevalence varied, with higher rates observed in day-care children (6.2%) compared to older than 5 years (1.2%) [17, 26]. In contrast, significantly higher MRSA colonization rates have been observed in children older than 5 years in Tanzania (10.5%) and Ethiopia (9.8%) [24, 27].

Resistance to erythromycin and clindamycin among *S. aureus* isolates was observed at 33.5% and 20.5%, respectively, suggesting the presence of diverse macrolide resistance mechanisms, a trend consistent with observations in other Latin American countries [16]. While clindamycin remains an option for treating *S. aureus* skin and soft tissue infections, its clinical utility is limited by emerging resistance, particularly inducible clindamycin resistance [28, 29]. Notably, the *D*-test revealed a high prevalence of inducible clindamycin resistance (20.5%) in our study, emphasizing the importance of routine *D*-test performance in clinical microbiology [30]. This finding raises concerns about the empirical use of clindamycin [24, 31]. In contrast, no resistance to trimethoprim-sulfamethoxazole was observed in our study, highlighting the continued importance of ongoing local antibiotic susceptibility surveillance to guide appropriate clinical management, especially considering the high resistance rates to trimethoprim-sulfamethoxazole (30%–60%) reported in Africa [24].

The observed pneumococcal colonization frequency (20.2%) aligns with previously reported rates in low- and middle-income countries [8, 32]. This frequency is slightly lower than observed in pre-PCV7 era studies in the region [10, 33], potentially reflecting the impact of PCV vaccination on reducing carriage of vaccine serotypes [8]. However, our study also found an association between PCV13 vaccination and increased pneumococcal colonization, primarily by non-PCV13 serotypes, suggesting potential selective pressure exerted by the vaccine [9]. Consistent with previous findings [33–35], factors associated with increased pneumococcal colonization included mild respiratory illness, living with other children, and younger age. Of note, hospitalization within 3 months prior to sample collection was associated with a decreased prevalence of pneumococcal colonization (aPR: 0.29; 95% CI: 0.09–0.99; *p* = 0.048), potentially due to recent antibiotic treatment in hospitalized children [9].

Macrolide resistance in *S. pneumoniae* isolates was observed at 48.9%, a significant increase compared to pre-PCV7 era findings in the same population [33]. Molecular analysis from our previous studies [9] revealed that the macrolide resistance mechanisms were primarily mediated by the *erm*(B) gene (ribosomal methylation) and the MEGA element (*mef*A/E) (macrolide efflux pump).

The observed co-colonization frequency of 2.9% was comparable to that reported in a previous study among Peruvian children in Cajamarca (4.8%) using microbiological assays [10]. However, this rate is lower than observed in studies using molecular methods (28.2%), which generally exhibit higher sensitivity in detecting potential nasopharyngeal pathogens [10]. Care should be taken with prior antibiotic use, as DNA detection from non-viable bacteria may not indicate current colonization [36]. An inverse association was found between *S. pneumoniae* and *S. aureus* colonization. These two species, while both colonizing the nasopharynx, may exhibit competitive interactions [4, 5, 37]. For instance, some *S. pneumoniae* strains can produce competence-stimulating peptides that inhibit *S. aureus* biofilm formation [38], while others produce H_2_O_2_, which can eradicate *S. aureus in vitro* [39]. However, the impact of this interaction may vary depending on the age of the child, with competition potentially being less pronounced in older children [40].

Our study had some limitations. Firstly, while *S. pneumoniae* strains were isolated promptly, *S. aureus* isolation was delayed (8–12 months). Although nasopharyngeal swabs were stored at −80 °C in STGG medium to maintain viability, this delay may have introduced potential biases. Secondly, the study relied on convenience sampling, potentially limiting the generalizability of the findings beyond the specific population of children attending public hospitals in Lima and Callao. While this population represents a significant proportion (27.2%) of children under 2 years old in Peru, it may not fully reflect the characteristics of children seeking care in private healthcare facilities or residing in other regions. Moreover, there is insufficient evidence to conclude an association with the co-colonization status, as it was an infrequent event, which may have affected the statistical power for this category; therefore, we recommend interpreting the results for this group as exploratory. Finally, limitations in immunization data collection, including incomplete records and the inability to assess adherence to the national immunization schedule, may have influenced the study findings.

Despite the introduction of three different PCVs into Peru’s National Immunization Program, the country lacks population-level surveillance of emerging bacterial pathogens, a crucial step following vaccine introduction, as exemplified by post-Hib vaccine surveillance [41]. This study highlights the need for continued surveillance of nasopharyngeal pathogens, such as *S. pneumoniae* and *S. aureus*, particularly given the increasing prevalence of antimicrobial resistance. Our findings suggest that promoting exclusive breastfeeding may contribute to a reduction in *S. aureus* colonization. Furthermore, while PCV13 vaccination showed an association with decreased *S. aureus* colonization, it was also associated with increased *S. pneumoniae* colonization, potentially due to selective pressure on non-vaccine serotypes. These findings emphasize the importance of ongoing surveillance to monitor the impact of PCVs on the nasopharyngeal microbiota and the emergence of potential vaccine-related consequences.

## Supporting information

10.1017/S0950268825100277.sm001 Li Valverde et al. supplementary material Li Valverde et al. supplementary material

## Data Availability

The raw data supporting the conclusions of this article will be made available by the authors, without undue reservation.

## Supplementary material

The supplementary material for this article can be found at http://doi.org/10.1017/S0950268825100277.
