# Supplementary material for: Antibiotic resistance and factors associated with colonization dynamics of Staphylococcus aureus and Streptococcus pneumoniae in healthy children in Lima, Peru
Source: Epidemiol Infect. 2025 Sep 4;153:e106. doi: 10.1017/S0950268825100277 (PMC12455507; doi:10.1017/S0950268825100277)
Supplement: Li Valverde et al. supplementary material [file S0950268825100277sup001.docx]

**Figure S1: Directed Acyclic Graph (DAG)**
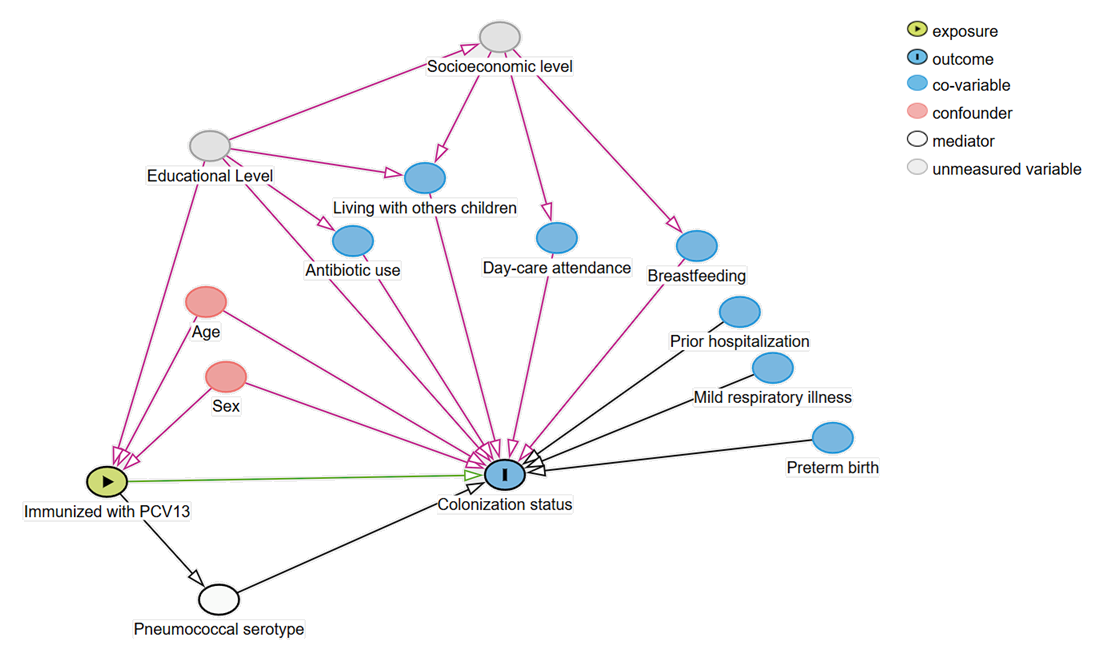


**Table S1. Association between *S. aureus* and *S. pneumoniae* vaccine and non-vaccine serotype**

| **Characteristics** | | **Colonization status** | | |  | **Pneumococcal colonization** | | | | | | |
| --- | --- | --- | --- | --- | --- | --- | --- | --- | --- | --- | --- | --- |
|  |  | ***S. aureus* colonizated** | | |  | **Non-vaccine serotypes** | | |  | **Vaccine serotypes** | | |
|  |  | **PR** | **IC 95%** | **p** |  | **PR** | **IC 95%** | **p** |  | **PR** | **IC 95%** | **p** |
| *Streptococcus pneumoniae* colonization | |  |  |  |  |  |  |  |  |  |  |  |
|  | Non-colonizated | Ref. |  |  |  | - |  |  |  | - |  |  |
|  | Colonizated | 0.68 | 0.68 - 0.99 | 0.047 |  | - | - | - |  | - | - | - |
|  |  |  |  |  |  |  |  |  |  |  |  |  |
| *Staphylococcus aureus* colonization | |  |  |  |  |  |  |  |  |  |  |  |
|  | Non-colonizated | - |  |  |  | Ref. |  |  |  | Ref. |  |  |
|  | Colonizated | - | - | - |  | 0.69 | 0.44 - 1.06 | 0.087 |  | 0.70 | 0.28 - 1.79 | 0.461 |

Bivariate analysis using robust (modified) Poisson regression.

PR: Prevalence ratio. 95 % CI: 95 % confidence interval. Ref: Reference category.
